# Regulating the Practice of Medicine in Colorado

**Published:** 1884-03

**Authors:** 


### Regulating the Practice of Medicine in Colorado.

The law which results in the survival of the fittest may yet be generally recognized as preëminently efficient for the regulation of medical practice. In a civilized community the victim of the charlatan dies, and the guilty man is merely handed over to the almost impotent arm of a secular law, which is hampered greatly by the orthopœdic devices strapped to it by the ingenuity of "Sanitarians."

"They do these things better in" Colorado, according to the subjoined item which we find in one of the daily journals :

FREEPORT, ILL., Feb. 18.—News has been received here of the hanging, by a mob, near Denver, Col., of Eli Madlong, a former resident of Freeport. It seems that Madlong pretended to be a physician, although he had no medical education whatever, and was nothing but a quack. To one of his patients he gave a dose that killed him. The victim's friends organized a vigilance committee and hanged the man to prevent any further deaths at the pretended doctor's hands. Madlong was known here as a hard character. His father, a tailor by trade, still lives here, and is a nice old man.

---

THE French Academy and the French journals having discussed in a more or less excited manner the question of cold applications in typhoid fever for over a year, they are now in a heated debate over the stimulant action of beer and its use in hospitals. The director of public assistance in Paris has begun the attack with an order restraining the surgeon of hospitals from prescribing beer as a common tonic. The surgeons have declared war against this order and they have so far their own way.

ON account of the departure of the senior editor, Dr. Pilcher, for Europe, in January, and the expected departure of his colleague, Dr. Fowler, for a similar trip abroad, later in the season, the publication of the *Annals of Anatomy and Surgery* is necessarily suspended during their absence.

If circumstances shall seem to warrant the resumption of the journal with the beginning of another year, due notice will be given.

THE friends and admirers of Mr. Jonathan Hutchinson, F.R.S., of the London Hospital, have subscribed a fund for the foundation of a Hutchinson prize essay for clinical surgery, to be awarded every three years to members of the hospital of not more than ten years' standing from entrance.

